# Intestinal iron bio-accessibility changes by Lignin and the subsequent impact on cell metabolism and intestinal microbiome communities†

**DOI:** 10.1039/d2fo03807b

**Published:** 2023-03-21

**Authors:** Richard D. Horniblow, Prachi Pathak, Maryam Eshrati, Gladys O. Latunde-Dada, Chris Tselepis

**Affiliations:** a School of Biomedical Sciences, University of Birmingham Edgbaston Birmingham B15 2TT UK r.horniblow@bham.ac.uk +44 (0)121 415 1991; b Department of Nutrition and Dietetics, Kings College London Franklin-Wilkins Building Stamford Street London SE1 9NH UK

## Abstract

The detrimental effects of high concentrations of colonic iron have been linked to intestinal inflammation and microbial dysbiosis. Exploiting chelation against this *luminal* pool of iron may restore intestinal health and have beneficial impacts on microbial communities. This study aimed to explore whether lignin, a heterogenous polyphenolic dietary component, has iron-binding affinity and can sequester iron within the intestine and thus, potentially modulate the microbiome. Within *in vitro* cell-culture models, the treatment of RKO and Caco-2 cells with lignin almost abolished intracellular iron import (96% and 99% reduction of iron acquisition respectively) with corresponding changes in iron metabolism proteins (ferritin and transferrin receptor-1) and reductions in the labile-iron pool. In a Fe-59 supplemented murine model, intestinal iron absorption was significantly inhibited by 30% when lignin was co-administered compared to the control group with the residual iron lost in the faeces. The supplementation of lignin into a microbial bioreactor colonic model increased the solubilisation and bio-accessibility of iron present by 4.5-fold despite lignin-iron chelation previously restricting intracellular iron absorption *in vitro* and *in vivo*. The supplementation of lignin in the model increased the relative abundance of *Bacteroides* whilst levels of *Proteobacteria* decreased which could be attributed to the changes in iron bio-accessibility due to iron chelation. In summary, we demonstrate that lignin is an effective luminal iron chelator. Iron chelation leads to the limitation of intracellular iron import whilst, despite increasing iron solubility, favouring the growth of beneficial bacteria.

## Abstract 2

Lignin chelates iron within the gastrointestinal lumen, altering bio-accessibility and leading to modulated enterocyte iron metabolism and changes in intestinal bacteria.

## Graphical abstract

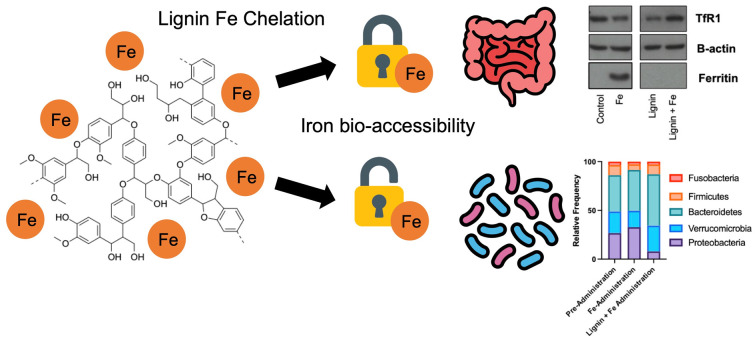

## 1. Introduction

Iron is a vital micronutrient as it is utilised in many metabolic processes, including oxygen transport, DNA synthesis and cell-cycle progression.^1^ As such, to sustain body iron stores of around 35 and 45 mg kg^−1^ for adult women and men respectively, the recommended daily intake of iron is around 8.7 mg for men and 14.7 mg for women. It is reported, however, that the average daily consumption of iron is approximately 20 mg. This is largely attributed to the increased consumption of westernised diets which are high in red meat and processed foods, in addition to iron-fortified foods including cereals and bread.^2^ This high dietary intake of iron, alongside a limited rate of small intestinal absorption (0.7 to 22.9%) accounts for the relatively high concentration of iron within the colonic lumen.^3^ High concentrations of systemic iron need to be tightly regulated since free iron can catalyse reactive oxygen species (ROS) production leading to lipid peroxidation and ferroptosis, commonly occurring in degenerative diseases such as hereditary hemochromatosis. In contrast, the luminal pool of free iron in the colon is not regulated and its chemical reactivity can pose potential intestinal health risks. The ROS-generating capacity of high levels of iron within the faeces due to ferrous sulphate supplementation has been reported.^4^ Iron supplementation is known to impact the intestinal microbiome and induce microbial dysbiosis.^5^ Jaeggi *et al.* demonstrated that iron fortification in a group of Kenyan infants induced intestinal inflammation that was associated with the increased abundance of pathogenic bacteria which caused gastrointestinal disease.^6^ Ferrous sulphate supplementation has also been demonstrated to cause significant changes in mouse caecal microbial communities.^7^ A meta-analysis concluded that ferrous sulphate supplementation is associated with a significant increase in gastrointestinal side-effects, including constipation, nausea and diarrhoea,^8^ despite ferrous sulphate being a first-line treatment for iron deficiency anaemia (IDA) which affects 2–5% of adult men and postmenopausal women in the UK.^9^ In gastrointestinal inflammatory conditions such as inflammatory bowel disease (IBD), more than half of such patients will reduce their dose or withdraw from iron supplementation due to poor tolerability.^10,11^ Consequently, ferrous sulphate supplementation is associated with gastrointestinal toxicity, whether it be through exacerbation of inflammation, changes in microbial communities or a combination of both.

Iron-chelation strategies have therapeutic utility in systemic iron overload, and thus, iron chelation directed at the colon may have health benefits if this luminal pool of iron is having toxic effects; depletion of luminal iron has been demonstrated to be beneficial.^7^ Targeting iron chelation within the colon has been explored with the use of non-absorbable biopolymeric fibre-like iron chelators.^12^ However, such fibres are commonly fermented by the microbiome and hence the bioactivity and iron-chelating properties are severely compromised.^13^ Moreover, many other nutraceutical-like compounds (for example polyphenolic compounds and tannins) have been reported to have iron-chelating properties and here, we highlight the possibility of the natural biopolymer lignin acting as a novel colonic iron chelator.

Lignin is a highly un-ordered, aromatic, cross-linked and polyphenolic polymer which provides structural rigidity in plant cell walls.^14^ It is categorised as a natural dietary fibre, similar to cellulose, hemicellulose and pectin and is considered indigestible and inert.^15^ Daily average intakes of lignin are unknown, yet, as lignin is the second most abundant natural biopolymer behind cellulose, and accounting for 15–25% (w/w) of herbaceous biomass, its consumption is considerable.^16^ In a human study, lignin was found to be undigested in both the small and large bowel^17^ though, conversely, there is also evidence that lignin can be fermented by the intestinal microbiome to smaller molecular weight compounds in the large bowel.^18,19^ Whether these lignin-derived metabolites or degradation components retain any iron chelating properties remains undetermined.

In this study, we aim to establish if lignin has iron chelating properties in a variety of cellular, bacterial, and *in vivo* models to (i) confirm if lignin has iron binding affinity, (ii) examine how lignin alters cellular iron absorption and metabolism, and (iii) determine if iron chelation within a gut microbiome model alters bacterial communities. These results will provide further insight into the digestive properties and consequences of lignin consumption.

**Hypothesis:** Lignin has iron chelation ability within the colon and could alter the negative impacts of high levels of gastrointestinal iron.

## 2. Materials & methods

### 2.1 Materials, chemicals, and reagents

Caco-2 (HTB-37) and RKO (CRL-2577) cells were obtained from the ATCC (Manassas, USA). The antibodies utilised in this study were obtained from; Ferritin light-chain (Abcam AB69090, 1 : 5000 dilution), β-actin (Abcam AB8226, 1 : 5000 dilution) and Transferrin Receptor 1 (TfR1) (Invitrogen, H68.4, 1 : 1000). Radiolabelled ^59^FeCl_3_ and OPTIPHASE HISAFE3 scintillation fluid were obtained from PerkinElmer (Buckinghamshire, UK). CD1 mice were purchased from Charles River (UK). Streptomycin and the Peirce BCA kit (#23225) were obtained from ThermoFisher Scientific (Paisley, UK). Calcein AM and 6-well Transwell inserts were obtained from Corning (Flintshire, UK). Nutritional feed for the intestinal bioreactors was obtained from ProDigest (Ghent, Belgium). All other chemicals were obtained from Sigma Aldrich/Merck (Dorset, UK). The ferrous sulphate (FeSO_4_·7H_2_O) and Lignin were obtained from Sigma Aldrich/Merck (Dorset, UK).

Lignin [Lignin Alkali (Product 370959, Sigma Aldrich)] was extracted from wood pulp (derived from Norway spruce) and processed under alkali conditions to exclude cellulose contamination. This method of hot alkali extraction produces lignin that is soluble in aqueous conditions. The chemical structure of lignin is complex and heterogenous throughout nature. A chemical description and representation of lignin and its heterogenous nature has been described elsewhere.^16,20^

### 2.2 Cell culture

Caco-2 and RKO cells were routinely cultured in DMEM with FCS (10% v/v), penicillin (50 U mL^−1^) and streptomycin (50 μg mL^−1^). RKO cells are a poorly differentiated colon carcinoma lineage. Caco-2 cells are an excellent cell line of the small intestine as, under the correct conditions (see Section 2.7), spontaneously differentiate into a small-intestinal monolayer.

Cells were seeded into 6-well plates at a concentration of 1 × 10^5^ cells per mL with 2 mL of medium added per well. In experiments with iron incubation, a standard protocol was employed. The form of iron used was FeSO_4_ at a final concentration of either 10 or 100 μM. Sodium ascorbate was also included at either 50 or 500 μM, ensuring a 1 : 5 ratio of iron to sodium ascorbate. To create an iron and lignin-containing medium, lignin alkali (2% w/v in deionized H_2_O) was mixed with DMEM cell growth medium with iron (at either 10 or 100 μM) to create a resultant lignin (0.3% w/v) with iron medium. Cells were cultured with the supplemented media for 24 h. Cells were also cultured with media only, this ‘no treatment’ group represented the control. After this period, the media was removed, and cells were washed with PBS and cells harvested as detailed within the individual experimental methods.

### 2.3 Equilibrium dialysis

Aqueous lignin solutions (0.1% w/v, 5 mL) were sealed into a dialysis membrane (*M*_w_ cut off = 12 400 Da) and incubated in aqueous FeSO_4_ in DI H_2_O (10 mM, 1500 mL) for 24 h and subsequently washed in DI water for 120 min. To measure iron that would enter the dialysis membrane as a result of diffusion only (the experimental control), DI H_2_O (5 mL) was also sealed and incubated within the iron solution and similarly washed. Following this, the concentration of iron in samples within the dialysis membrane was determined using the ferrozine assay.

### 2.4 Quantification of cellular iron

Post iron co-culture in the presence or absence of lignin, cells were lysed in 150 μL HEPES-saline lysis buffer (10 mM, pH 7.4 with NaCl 0.9% w/v). A ferrozine stock reagent of sodium acetate (17 mM) l-sodium ascorbate (4.6 mM) and 3-(2-pyridyl)-5,6-diphenyl-1,2,4-triazine-*p*,*p*‘-disulfonic acid monosodium salt hydrate (0.18 mM) was prepared in DI H_2_O. The cell lysate was thoroughly mixed and 90 μL was aspirated and mixed with 200 μL trichloroacetic solution (20% w/v), which was then heated at 100 °C for 10 min and afterwards centrifuged at 12 000 rpm for 5 min to pellet the protein precipitate. The supernatant was aspirated and 200 μL was added to 600 μL ferrozine stock solution and mixed thoroughly and the absorbance read at *λ* = 550 nm. All results were standardised to protein content using a protein assay kit.

### 2.5 Quantification of the cellular labile iron pool

The iron-chelating intracellular dye (Calcein-AM) was employed to examine the labile iron pool. Cells were cultured with Calcein AM (0.0625 μM) for 15 min at 37 °C in PBS. Calcein-AM was removed from the cells, washed twice with PBS and the cells were subsequently cultured with either lignin and/or FeSO_4_ (100 μM). At the end of the culture period, media was removed, cells were washed twice with PBS, trypsinised and centrifuged (600 rpm, 5 min). The cell pellet was re-suspended in PBS and re-centrifuged. The cell pellet was then finally re-suspended in PBS (150 μL) and analysed for mean fluorescence intensity in FL-1 on an Accuri-C6 flow cytometer (BD Biosciences, Berkshire, UK). As Calcein-AM fluorescence is quenched upon iron chelation, the higher the concentration of the LIP, the lower the fluorescence intensity; therefore, the fluorescence is inversely proportional to LIP concentration. Consequently, the data presented from LIP measurements are provided as 1/FL-1 fluorescence intensity.

### 2.6 western blotting

Cells cultured with lignin and/or iron were lysed in RIPA lysis solution (containing (4-Nonylphenyl poly(ethylene glycol) (1% w/v), sodium deoxycholate (0.5% w/v), sodium dodecyl sulphate (0.1% w/v) in DI H_2_O) on ice. Cell lysates were sonicated for 5 s and protein concentrations were determined using a protein assay. Cell lysates were then subject to western blotting as previously described.^21^

### 2.7 Caco-2 Transwell studies

Caco-2 cells were seeded into pre-treated collagen-coated 6-well Transwell inserts at a concentration of 4 × 10^5^ cells per mL. Cells were grown for 20 days post-confluency when a constant trans-epithelial electrical resistance value of 400 Ω cm^2^ was sustained. Prior to co-culture with lignin and/or iron, the cell medium was changed to FCS-free MEM and cells were subsequently treated with iron, FeSO_4_ spiked with Fe-59 and/or lignin (0.3%). To prepare Fe-59 spiked iron media, a stock solution (40 mL) of aqueous FeSO_4_ (10 mM) and sodium ascorbate (500 mM) was spiked with ^59^FeCl_3_ to reach a radiation concentration of 10 000 counts per minute per well. This stock was diluted into the media to prepare the 100 μM FeSO_4_ solution as detailed earlier. To the apical chamber, FCS-free MEM with Fe-59 spiked iron with or without lignin (2 mL) was added. Media samples from the basolateral chambers were removed over the time course and after 24 h, media were removed, and the cells were washed with Versene (0.2 g L^−1^ EDTA in PBS) and lysed in RIPA buffer. Samples collected were assessed for iron concentration using scintillation counting. Lysates collected were transferred into scintillation tubes containing scintillation fluid (1 mL, OPTIPHASE HISAFE3) and counted on a gamma counter (Packard 2500 TR liquid scintillation counter, PerkinElmer).

### 2.8 Animal studies

All *in vivo* experiments were carried out under Home Office approved conditions and animal care and the regulation of scientific procedures met the criteria laid down by the United Kingdom Animals (Scientific Procedures) Act 1986. All animal procedures were performed in accordance with the Guidelines for Care and Use of Laboratory Animals of King's College London, and experiments were approved by the Animal Welfare and Ethical Review Bodies (AWERB) of King's College London.

Male, 6-week-old, CD1 mice were used in ^59^Fe absorption experiments and mice were given access to water and food *ad libitum.* A ^59^Fe-spiked iron gavage solution was prepared by dilution of a Fe(ii) stock solution into HEPES buffer (HEPES, 16 mM, pH 7.4, NaCl 125 mM) to a concentration of 200 μM Fe(ii) and 1000 μM sodium ascorbate. All mice were given an iron-deficient diet for 6-weeks prior to experimentation. Administration of lignin (2% w/v in DI H_2_O, 100 μL) to half of the mice was performed immediately after gavage of the prepared radio-labelled iron (100 μL), whereas the other half received an iron-only gavage and no lignin. All mice were housed in metabolic chambers for 24 h prior to culling to allow the collection of faecal samples. After 24 hours, mice were culled and dissected. Detection of iron concentrations was performed as previously reported.^12^ Carcass counts per minute measurements were performed on a whole-body animal counter (LIVE-1 Technical Associates, CA, USA).

### 2.9 Intestinal bioreactor setup and Lignin administration

We established a faecal inoculated *in vitro* model of human colonic microbiota (MIMIC, Model of the Intestinal MICrobiome) which was based on previous *in vitro* gastrointestinal simulation.^13^ In brief, the model consisted of three parallel faecal-inoculated bioreactors (Electrolab, Tewkesbury, UK) enabling the experiment to be performed in triplicate. At the start, a total volume of 300 mL nutritional feed media containing a mixture of carbohydrate-based medium and simulated pancreatic fluid which was composed of dehydrated bile (6.0 g L^−1^), NaHCO_3_ (12.5 g L^−1^) and porcine pancreatin (0.9 g L^−1^) was added to each vessel and continuously mixed by a magnetic stirrer. The temperature of each vessel was stabilised individually at 37 °C using a temperature controller (Electrolab, Tewkesbury, UK). The pH was also maintained by controllers between the set limits of pH 6.15–6.8 through automated titration of HCl (0.5 mol L^−1^) or NaOH (0.5 mol L^−1^). The complete system was maintained under a nitrogen atmosphere.

To inoculate the bioreactors with an intestinal microbial community, a faecal sample donated from a single healthy volunteer was utilised. A fresh faecal sample (10 g) was diluted and homogenized with 50 mL sterilised phosphate buffer (K_2_HPO_4_, KH_2_PO_4_, 0.1 mol L^−1^, pH 6.8), containing sodium thioglycolate (0.5 g L^−1^). After the removal of the particulate material by centrifugation (500*g*, 1 min), the supernatant (12.5 mL) was introduced into each vessel. After this point, the bioreactors were equilibrated with no further adjustments to stabilise the microbial communities.

Throughout the experimental procedure, 20% of the total contents were removed and replaced with fresh nutritional feed media in the morning (AM) and the evening (PM) every day for a period of 30 days. Each vessel was flushed before and after feeding for 10 min with oxygen-free N_2_ gas to ensure anaerobic conditions.

#### 2.9.1 Iron supplementation

On day 10, aqueous FeSO_4_ (10 mM) containing sodium ascorbate (50 mM) was introduced into the vessels to evolve the iron concentration to 100 μM over three consecutive days. Thereafter, the iron concentration within the bioreactors was maintained at 100 μM. Iron supplementation was included within the AM/PM feeding periods.

#### 2.9.2 Lignin supplementation

After 10 days of iron supplementation, lignin was also co-administered (with iron) by injecting an aqueous lignin solution to maintain bioreactor lignin concentrations at 0.3% (w/v) in each vessel. Lignin co-administration with iron was included within the AM/PM feeding periods. A schematic of the experimental phases and treatment schedule of the bioreactors is detailed in Schematic 1 (ESI†). Sampling aliquots were withdrawn before each feeding addition to assess iron content and modification of colonic microbiota composition.

### 2.10 Bioreactor iron assessments

The *total iron* content of the bioreactor slurry was assessed using the ferrozine assay. A ferrozine stock reagent, as prepared earlier, was utilised. In brief, 200 μL of bioreactor sample was mixed with a trichloroacetic acid solution (200 μL, 20% w/v), which was then heated at 100 °C for 10 min and then centrifuged (12 000 rpm, 5 min) to pellet the protein precipitate. The supernatant was aspirated and 200 μL was added to 600 μL ferrozine stock solution and mixed thoroughly and the absorbance was read at *λ* = 550 nm.

To quantify the amount of *free iron*/*non-soluble iron*, 1 mL of bioreactor sample was centrifuged (12 000 rpm, 5 min) and 200 μL of the supernatant aspirated for analysis with the ferrozine reagent.

To evaluate the *exchangeable iron*, 200 μL of the bioreactor sample was treated with 200 μL of TE buffer (10 mM Tris, 1 mM EDTA) *via* continuous mixing for 10 min at room temperature. Following this, samples were centrifuged (12 000 rpm, 5 min) and 200 μL of supernatant was subjected to ferrozine assay.

### 2.11 Bacterial DNA extraction and sequencing

Bioreactor samples (2 mL) were centrifuged (12 000 rpm, 5 min) and bacterial DNA was extracted from the resultant cell pellet using QIAamp DNA Stool Mini Kit including mechanical lysis using the TissueLyser II (QIAGEN) as previously reported.^22^ DNA concentration was quantified using a Qubit dsDNA BR assay kit (Invitrogen) and diluted to the concentration of 1 ng μL^−1^. The Earth Microbiome Project Protocol was employed to prepare the sequencing library. Paired-end sequencing (2 × 250 bp) was performed on the Illumina MiSeq platform (Illumina).

### 2.12 QIIME 2 diversity analysis and taxonomic composition 16s rRNA sequence analysis

Sequencing results were processed using Quantitative Insights into Microbial Ecology 2 (QIIME2, v.2022.02).^23^ Paired-end demultiplexed sequences were imported into QIIME prior to quality filtering. A total of 2 081 032 demultiplexed sequence counts were generated from the DNA samples obtained from the bioreactor sampling. The sequences were denoised using the dada2 plugin (*via* q2-dada2).^24^ A rarifraction value of 30 000 sampling depth was used. Diversity analysis was undertaken to assess within-sample diversity (alpha diversity) and between-sample diversity (beta diversity). Faith's phylogenetic diversity (PD) metric was employed to examine bacterial community richness whilst incorporating phylogenetic relationships between taxa.^25^ Faith's PD illustrates the microbial richness and diversity within samples. Faith's PD is a qualitative measure of community richness that incorporates phylogenetic relationships between the features. Differences were statistically tested using the Kruskal–Wallis method. Beta diversity analysis (changes between samples) was undertaken using weighted UniFrac and unweighted UniFrac.^26^ Principal Coordinates analysis plots were generated for both unweighted and weighted UniFrac data to visualize the microbial communities. Taxonomy was assigned to ASVs using the q2-feature-classifier classify-sklearn naïve Bayes taxonomy classifier against the Greengenes 13_8 99% OTUs reference sequences.^27,28^ Differential taxa analysis was identified *via* the Linear Discriminant Analysis (LDA) using LEfSe which employed the Kruskal–Wallis test at a pre-defined *α* of 0.05.^29^ Significantly different relative abundances between groups were used for LDA.

## 3. Results

### 3.1 Lignin chelates with iron and limits cellular iron acquisition

**Fig. 1:**
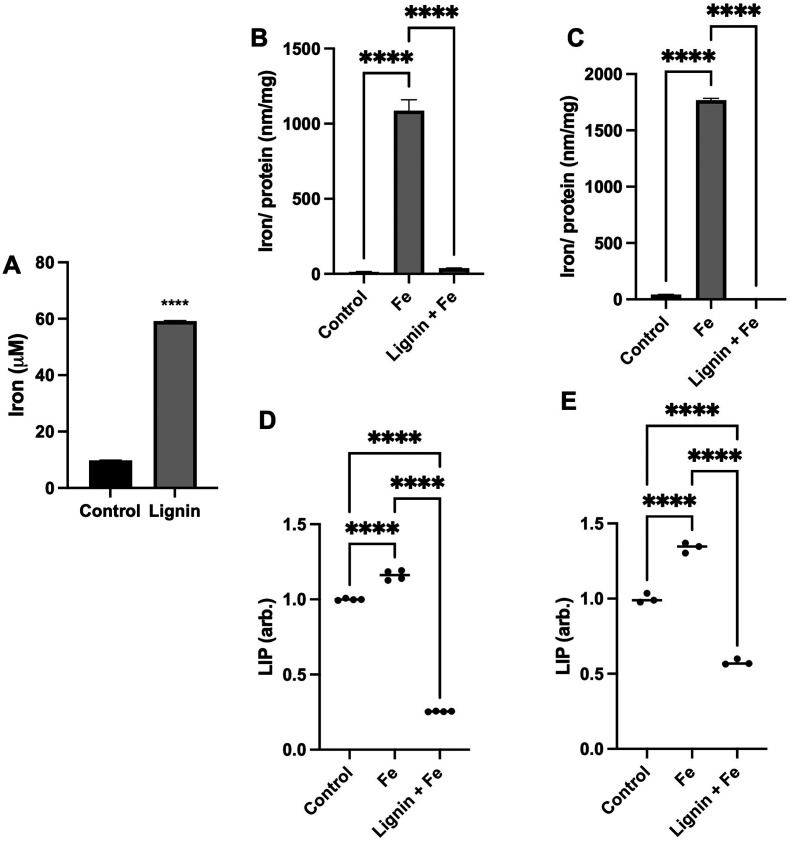
Iron chelation properties of lignin. (A) The iron binding capacity of lignin post equilibrium dialysis against iron with “Control” representing the concentration of iron within the dialysis tubing containing water only. (B) Intracellular iron concentrations of RKO and (C) Caco-2 cells exposed to iron in the presence and absence of lignin. Intracellular labile iron pool (LIP) concentrations of (D) RKO and (E) Caco-2 cells treated with iron in the presence and absence of lignin normalised to control. Data displayed as mean values (*n* = 3) with error bars representing ± standard deviation and **** denotes *p* < 0.0001. “Control” in these cell experiments (B–E) represents the non-treated group.

To quantify the capacity of iron binding to lignin, equilibrium dialysis was employed. Lignin did significantly demonstrate iron binding capacity (Fig. 1A) with a lignin : iron ratio of *ca.* 1 : 16 under these aqueous conditions. Lignin was able to bind 6× the concentration of iron that was measured due to diffusion into the water-only (control) dialysis membrane. To subsequently examine if lignin could chelate iron and directly influence intracellular iron concentrations under physiological conditions, RKO or Caco-2 intestinal cells were exposed to iron in the absence or presence of lignin (Fig. 1B and C). In both cell lines, treatment with iron only significantly increased intracellular iron concentration (81-fold and 41-fold for RKO and Caco-2 cells respectively, *p* < 0.0001 for all). Lignin + Fe treatment significantly decreased intracellular iron concentrations, reducing iron levels to those found in the control in both cell lines (*p* < 0.0001). Furthermore, this *in vitro* iron chelating effect was supported by examining the labile iron pool (LIP) in both cell lines (Caco-2: Fig. 1D and RKO: Fig. 1E). Lignin also inhibited cellular iron mobilisation into the LIP. Treatment of RKO and Caco-2 cells with iron alone raised the LIP concentration by 15.0% and 34.0% respectively (*p* < 0.0001). RKO and Caco-2 culture with iron and lignin inhibited this increase and also reduced the concentration of LIP below that of the control cells (75.0% and 43.0% below control for RKO and Caco-2 respectively, *p* < 0.0001).

### 3.2 Chelation of iron by lignin alters cellular iron metabolism

**Fig. 2:**
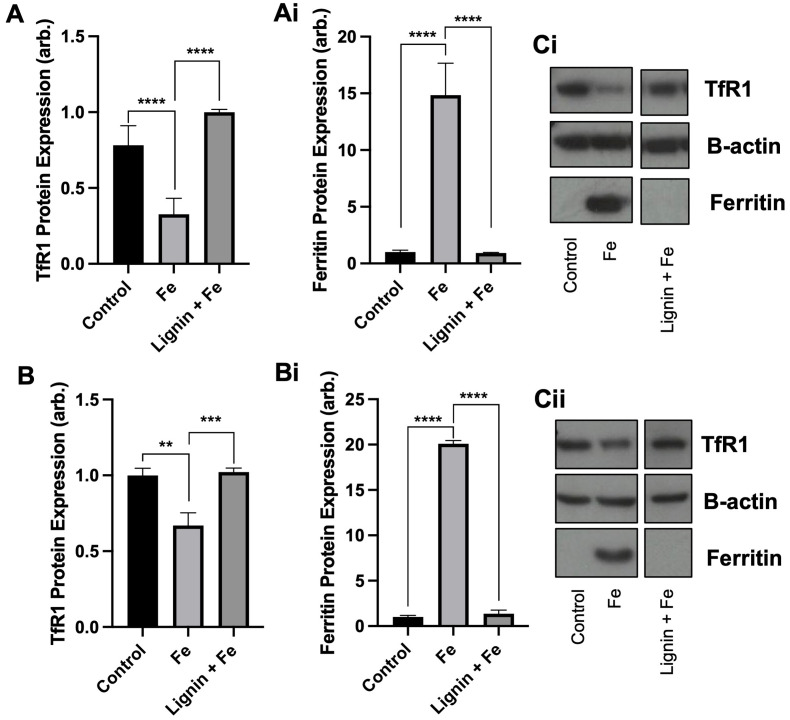
Cellular iron metabolism changes by lignin co-culture. (A) TfR1 and (Ai) ferritin protein expression within RKO cells and (B) TfR1 and (Bi) ferritin protein expression in Caco-2 cells cultured with iron in the presence or absence of lignin. Representative western blot images for (Ci) RKO and (Cii) Caco-2 cells. Data displayed as mean values (*n* = 3) with error bars representing ± standard deviation and **, *** and **** denoting *p* < 0.01, 0.005 and 0.0005 respectively. “Control” represents the non-treated group.

With the observation that lignin was able to chelate iron *in vitro* and restrict cellular iron acquisition, we next sought to examine if chelation of iron by lignin renders the iron non-accessible or utilisable by cells by examining both TfR1 and ferritin protein expression. Both TfR1 and ferritin protein expression are modulated by cellular iron concentrations. Ferritin is an iron storage protein, and, in conditions of high cellular iron, its expression will be increased to store this iron. In contrast, TfR1 is a receptor responsible for intracellular iron import and consequently, its expression becomes decreased in conditions of high cellular iron. We observed these changes in our cell lines treated with iron. Following the culture of RKO and Caco-2 cells with iron, TfR1 expression decreased by 58.3% (*p* < 0.005) and 33.1% (*p* < 0.01) and ferritin expression increased by 14-fold (*p* < 0.0005) and 20-fold (*p* < 0.0005) compared to controls for RKO and Caco-2 cells respectively (Fig. 2A and B). Subsequent iron co-culture in the presence of lignin demonstrated that lignin did indeed render iron non-accessible. TfR1 protein expression significantly increased to near-control levels for RKO and Caco-2 cells (*p* < 0.0005 and 0.005 respectively). Similarly, ferritin expression was diminished back to levels observed in control in both cell lines (*p* < 0.0005).

### 3.3 Lignin inhibits iron transport through a model epithelium membrane and impacts intestinal iron absorption in mice

**Fig. 3:**
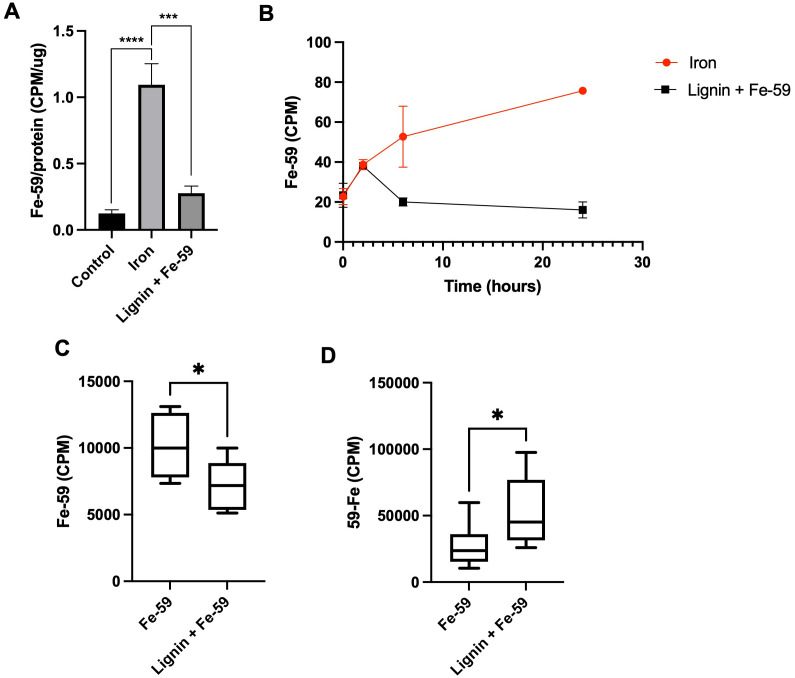
Changes in iron content across a cellular monolayer and in vivo due to lignin administration. (A) Intracellular Fe-59 Counts Per Minute (CPM, radiation activity originating from the radioactive source of Fe-59) within the Caco-2 monolayer post 24 hours exposure to iron in the absence or presence of lignin (*n* = 3). (B) Subsequent changes over the 24-hour incubation period within basolateral compartments of Fe-59 CPM in the absence or presence of lignin. (C) Dissected carcass and (D) Faecal concentrations of Fe-59 CPM post supplementation of Fe-59 gavage with lignin (*n* = 6) or without lignin (iron only, *n* = 8) administration. Faecal concentrations represent total faecal Fe-59 counts collected over the experimental period. Data displayed as mean values with error bars representing ± standard deviation and *, *** and **** denoting *p* < 0.05, 0.005 and 0.0005 respectively. “Control” represents the non-treated group.

To examine the iron chelation dynamics of lignin, a Caco-2 monolayer model and a murine model of cellular iron absorption were employed. A Caco-2 monolayer was exposed to Fe-59 in the presence or absence of lignin for a period of 24 h (Fig. 3A). After 24 hours of co-culture of Fe-59 alone, intracellular Fe-59 levels became elevated 8.9-fold with respect to control (*p* < 0.0005). This increase was diminished in the presence of lignin where intracellular Fe-59 concentrations increased by only 2.25-fold with respect to control and significantly reduced compared to iron alone treatment (*p* < 0.005). Throughout the culture period, the basolateral chamber was sampled (Fig. 3B). In the iron-only treated monolayers, iron concentration increased in the basolateral chamber as a function of time. In lignin and iron-exposed monolayers, this accumulation of iron within the basolateral compartment was not observed.

To examine iron chelation potential within the environment of the gastrointestinal tract, a murine model of iron absorption was employed. One murine cohort was administered an oral gavage of Fe-59 (*n* = 8) whereas, in a second cohort, this was followed by an immediate subsequent oral gavage of lignin (*n* = 6). Following a period of 24 hours, carcass (Fig. 3C), and faecal Fe-59 (Fig. 3D) concentrations were determined. Carcass iron concentrations were significantly decreased in the lignin with iron-administered cohort compared with the iron-only group (*p* = 0.023). This finding was reversed for the 24-hour faecal collections (Fig. 3D), where significantly increased levels of Fe-59 were found in the faeces of mice given lignin with iron (*p* = 0.037).

### 3.4 Lignin chelates iron in an intestinal bioreactor model and alters the proportion of iron in discrete bio-accessible pools

**Fig. 4:**
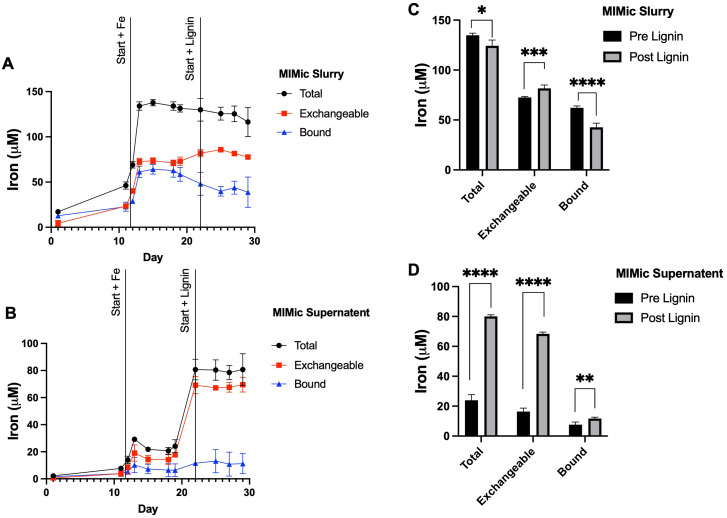
MIMic supplementation with iron and lignin. (A) Time-course measurement of total, exchangeable and bound iron within complete native MIMic slurry. (B) Time-course measurement of total, exchangeable and bound iron within MIMic suspension supernatant. Measurements in both cases were obtained over the periods of pre-administration, iron supplementation and lignin with iron supplementation phases. Average concentrations of total, exchangeable and bound iron were measured across the periods of iron and lignin and iron supplementation are presented for (C) MIMic slurry and (D) MIMic supernatant. Data displayed as mean values (*n* = 3) with error bars representing ± standard deviation and *, **, *** and **** denoting *p* < 0.05, 0.01, 0.005 and 0.0005 respectively.

To further examine the influence of lignin within the gastrointestinal tract on iron status, a bioreactor model (MIMic) was employed. Sequentially, MIMic was first supplemented with iron for a period of 10 days; the concentration of iron within the bioreactors increased steadily to 129.4 ± 9.7 μM. After this, lignin was also introduced with iron at a concentration of 0.3% (w/v). This treatment period is summarised in ESI Schematic S1.† Throughout both periods, samples were collected: native MIMic slurry is non-processed and thus represents the complete bacterial bioreactor suspension (Fig. 4A). The MIMic suspension supernatant is an intestinal slurry that has been centrifuged to obtain a clear supernatant (Fig. 4B). In both slurry and supernatant, two pools of iron were measured: total iron (the amount of iron measured directly from the sample) and exchangeable iron (the amount of iron post-treatment with EDTA). Bound iron was estimated from the total minus exchangeable iron.

**Within the slurry:** throughout iron supplementation, total iron concentration remained constant (134.3 ± 4.3 μM). Exchangeable iron remained stable (59.0 ± 20.7 μM) and likewise, bound iron (49.8 ± 18.3 μM). Upon lignin supplementation, there was a negligible decrease (3.8%) in average total iron (124.4 ± 11.2 μM, *p* = 0.013). Exchangeable iron significantly increased (81.7 ± 3.8 μM average, *p* = 0.002) and bound iron significantly decreased (42.6 ± 10.5 μM, *p* = 0.0002) (Fig. 4C).

**Within analogous sample supernatant:** throughout iron supplementation, the average total (within the supernatant) iron concentration across the 10 days was relatively stable (22.0 ± 5.5 μM). This was similarly the case for exchangeable iron (14.7 ± 5.0 μM) and bound iron (7.2 ± 4.1 μM). Upon lignin supplementation, there was a large increase in average total iron (within the supernatant) (80.07 ± 7.2 μM, *p* < 0.00001), a large increase in the average concentration of exchangeable iron (68.4 ± 4.0 μM, *p* < 0.00001) and a small but significant increase in the average concentration of bound iron (11.68 ± 6.2 μM, *p* = 0.0073) (Fig. 4D).

### 3.5 Microbial communities change due to lignin supplementation

**Fig. 5:**
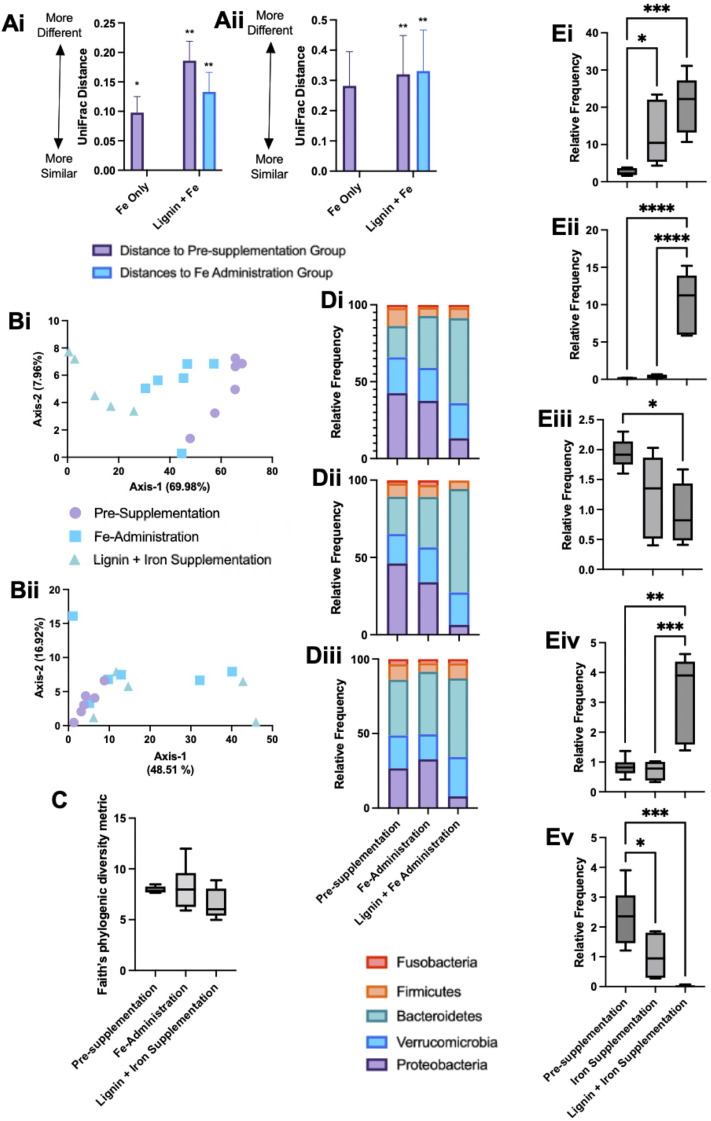
Microbial diversity and relative abundance frequency changes within colonic bioreactors. Mean (Ai) weighted and (Ai) unweighted UniFrac pairwise PERMANOVA distance comparison measurements between experimental phases. Respective Principal coordinate analysis (PCoA) plots of colonic bioreactor diversity based on weighted (Bi) and unweighted (Bii) UniFrac diversity metrics. (C) Faith's Phylogenic Diversity metric of bacterial species across the experimental phases. (D) Stacked phylum level bar plot of identified bacteria at phylum level between the pre-supplementation period, iron supplementation phase and iron with lignin supplementation phase groups; bar plots for each colonic-bioreactor vessel (V1–V3) are provided (Di–iii). (E) Average relative frequency changes across the three experimental phases within the three colonic bioreactors for (Ei) *Bacteroides uniformis*, (Eii) *Bacteroides ovatus*, (Eiii) *Biophilia*, (Eiv) *Clostridium and* (Ev) *Klebsiella.* Box plots represent the mean with ±SD and *, **, *** and **** denoting *p* < 0.01, 0.05, 0.005 and 0.0005 respectively.

Microbial communities were sampled from the bioreactors at the end of each experimental phase. DNA was extracted and sequenced and microbial changes with respect to alpha and beta diversity metrics were examined. Both unweighted UniFrac and weighted UniFrac distances were used to explore microbial compositional differences between the different experimental phases. Pairwise PERMANOVA distance measurements (Fig. 5A) demonstrate significant differences in microbial diversity between the groups. Weighted UniFrac distances demonstrated the greatest changes, with the lignin and iron phase significantly different from the pre-administration phase and the iron-only phase (Fig. 5Aii). The difference in microbial communities between the iron-only phase and the pre-supplementation phase was reduced in comparison to the diversity changes between the iron and lignin with the pre-supplementation phase. Overall, fewer diversity changes were observed with unweighted UniFrac (Fig. 5Ai). ESI Table S1† details the significance of these changes.

Principal coordinate analysis (PCoA) plots based on weighted (Fig. 5Bi), and unweighted (Fig. 5Bii) UniFrac distance metrics were generated. Weighted PCoA corroborated the diversity metrics sample populations clustering together. This was not the case for unweighted analysis, as expected as the diversity metrics obtained were smaller. For alpha diversity metrics, Kruskal–Wallis pairwise tests no significant changes in Faith's Phytogenic Diversity metric across any experimental phases (Fig. 5C).

The overall composition of the bioreactor microbiome was further analysed at the phylum (Fig. 5D) and deeper (Fig. 5E) taxonomic levels. Phylum level changes are detailed across the individual bioreactors (V1–3, Fig. 5Di–iii respectively). *Bacteroidetes* showed a tendency to increase across the experimental phases; this was verified at the species level with significant increases in *Bacteroides uniformis* and *Bacteroides ovatus* in the iron with lignin supplementation phase compared to the pre-supplementation phase (Fig. 5Ei and Eii). Also at a phylum level, the relative frequency of *Proteobacteria* decreased across the experiment with significant drops compared to pre-supplementation and with the iron with lignin supplementation phases (Fig. 5Di–iii). Further examination at a deeper level revealed a significant decrease in *Biophilia* (Fig. 5Eiii) and *Klebsiella* (Fig. 5Ev) in the iron with lignin supplementation phase compared to the pre-supplementation phase. *Clostridium* was also significantly increased in the iron and lignin supplementation phase (Fig. 5Eiv). These changes were validated using linear discriminant analysis of effect size (LEfSe) (data not shown).

## 4. Discussion

The primary objective of this study was to investigate the iron-binding properties of lignin and assess if this iron-binding potential has any physiological consequences on iron absorption in cell and mouse models and the iron status of the gastrointestinal environment of the colon. A secondary outcome of the study was to identify if any potential chelation effects attributed to lignin could alter microbial communities in the colon. With high levels of luminal iron associated with intestinal toxicity and microbial dysbiosis,^6,30^ the identification of potential colonic-acting iron chelators could be beneficial.

The iron chelating ability of lignin was first demonstrated through equilibrium dialysis experiments (Fig. 1A), where a ratio of interaction estimated to be 1 : 16 was found. Lignin is considered a highly complex biopolymeric chemical, constructed from three monolignols: *p*-coumaryl alcohol, coniferyl alcohol and sinapyl alcohol. These monomers themselves chemically have iron binding sites and thus multidentate binding with iron is highly likely. In both Caco-2 and RKO cell lines, culture in the presence of iron alone increased intracellular iron concentrations (Fig. 1B). Upon treatment with iron in the presence of lignin, the intracellular iron concentration significantly decreased, returning to levels found in the control. This suggests that lignin is binding iron within the extracellular medium and limits iron import across the cell membrane. This alteration of cellular iron status was further validated in both cell lines when the LIP was assayed (Fig. 1D & E). In both Caco-2 and RKO cells, iron administration significantly increased the concentration of iron within the LIP as expected. Subsequently, upon co-culture with lignin, LIP concentrations significantly decreased. The impact of lignin co-culture on cellular iron metabolism was examined through the quantification of ferritin and TfR1 protein expression (Fig. 2). In both Caco-2 and RKO cell lines, iron alone treatment significantly decreased TfR1 protein expression and significantly increased ferritin protein expression as expected. Upon exposure to lignin (in the presence of iron), these protein expression changes were reversed; lignin exposure re-established TfR1 protein expression and diminished ferritin expression. Together, these *in vitro* results indicate that lignin can chelate iron within a cellular setting and to our knowledge, these are the first reports of the impact of lignin culture on cellular iron status.

To further examine the iron chelation ability of lignin and its potential to act as a luminal iron chelator a Caco-2 Transwell model was employed. After 24 hours of iron-only treatment, intracellular levels of Fe-59 significantly increased within the Caco-2 monolayers (Fig. 3A); this increase was not found when lignin was present alongside iron, again suggesting the inhibition of cellular iron absorption by lignin. Following this, the flux of iron across the membrane, into the basolateral compartment was quantified (Fig. 3B). Throughout the iron-only treatment, iron increasingly accumulated within the basolateral compartment suggesting cellular export of Fe-59 following absorption. However, in the presence of lignin, after 2 hours of co-culture, no iron accumulation was found in the basolateral compartment, indicating a restriction of iron transport through the cell monolayer. Not only do these results validate the earlier observations of iron chelation, but they also indicate that the lignin-iron complex is itself non-absorbable.

To further validate these observations *in vivo*, the influence of oral lignin administration on murine iron absorption was examined. Higher levels of Fe-59 were observed in the faecal collections of the lignin-administered group in comparison to the iron-only cohort (Fig. 3Cii). These observations illustrate that iron is chelated by lignin within the gastrointestinal tract, restricting cellular absorption of iron, which then leads to an accumulation of iron in the faeces. However, whether lignin is still complexed with iron within the faeces is unknown. Previous studies performed in chicks supplemented with a lignin diet found no conclusive impact on iron status,^31^ whereas those undertaken in simulated gastrointestinal conditions demonstrated binding of iron by lignin.^32,33^ On the basis that many iron chelating agents have anti-oxidant capacity, it could be predicted that lignin also has the ability to quench reactive oxygen species (ROS), particularly if these reactions were taking place within the gastrointestinal tract. We have no evidence of an antioxidant role of lignin, and this would be an important consideration in any future study.

To further understand these iron-lignin interactions and the impact this might have on the intestinal microbiome, a gastrointestinal-simulated environment using a bioreactor-based system (MIMic) supplemented first with iron and then lignin with iron was employed. During iron-only supplementation, the majority of iron within the bioreactors was non-soluble or indirectly bound to bioreactor feed components, as only 17% of this total iron was detectable in the supernatant. Upon administration with lignin, two effects were observed; (i) the amount of iron within the supernatant significantly increased, with 62% of the total iron now detected in the supernatant (up 45% compared to pre-lignin supplementation) and (ii) the iron within the supernatant became highly exchangeable, with no corresponding iron increase in the slurry. This suggests that lignin is binding with iron and either solubilising it or disaggregating iron away from interactions with other nutritional components. This result, however, seems paradoxical based on our cell and *in vivo* experiments, as this chelation of iron by lignin increased the exchangeable nature of iron but does not however result in increased cellular bioavailability. This could be due to the different physicochemical environments of the gastrointestinal tract and the unknown type and state of iron in the large bowel. The bioreactor models (which modelled the colon) demonstrated iron solubilisation whereas iron restriction was found to take place in the murine small bowel (where iron absorption takes place). Additionally, solubilisation by lignin does not necessarily result in increased cellular bio-accessibility, as, despite the lignin-iron complex increasing iron solubility, the lignin-iron complex may not be directly absorbable (similar to what we have demonstrated for other polyphenolic compounds).^21^

The state of iron present within the gastrointestinal tract is an important consideration, and these studies were conducted with ferrous sulphate (with sodium ascorbate as the counter ion) to keep iron within a soluble form. This is a common route to experimentally manipulate iron concentrations in models, yet how closely this resembles the form and type of iron in the colon is unknown. Moreover, the speciation and solubility of iron which has transited from the small intestine to the colon is equally unknown and extremely difficult to predict.^5^ The colonic microbiome itself could alter the state of iron by extracellular reductases.^34^ Different food components can additionally affect the valence state and solubility of iron.^35^ Despite this, the form and state of iron within the colonic models employed here would mimic the pH and anoxic environments of the colon, which would likely favour the ferrous form.^36^

We further examined whether these changes in MIMic iron concentrations, driven by chelation with lignin, could alter the microbial communities present. *Firmicutes*, *Bacteroides* and *Proteobacteria* were amongst the dominating bacteria which is representative of the colonic microbiome. There was also a considerable contribution by *Verrucomicrobia*; this could be due to the pro-mucin conditions employed in this model enabling mucin-degrading bacteria (such as *Akkermansia muciniphila*) to flourish.^37^ With respect to changes over the experimental period, we observed only a trend of decreased bacterial richness and diversity within experimental groups from the pre-supplementation, iron only and iron with lignin supplementation phases (Fig. 5C). this was estimated using Faith's Phylogenic Diversity Metric. Similar results were also found in *in vitro* microbial community-based studies using a known iron chelating agent (bathophenanthroline disulfonate), where depriving microbial communities of iron irreversibly reduced community richness.^38^

*Proteobacteria* were most abundant in the iron alone administration phase with levels of *Proteobacteria* then decreasing in the lignin and iron supplementation phase. An iron-rich environment in the gut is conducive to *Proteobacteria* as many species can oxidise iron for their metabolism.^36^ Therefore high levels of *Proteobacteria* are expected during iron supplementation. Following the administration of lignin, levels decreased, including *Biophilia* and *Klebsiella* (Fig. 5Eiii and Ev). This would suggest that lignin is again chelating iron but limiting its utilisation by the bacteria, despite the observed solubilisation effect of lignin. Previous studies have also demonstrated changes in microbial composition upon chelator supplementation.^39^ There are possible physiological consequences associated with this diminishment of *Proteobacteria* by lignin supplementation. A review investigating the association between an abnormal expansion of *Proteobacteria* and intestinal dysbiosis found that an increased prevalence of *Proteobacteria* is a potential diagnostic signature of dysbiosis and risk of disease.^40^ In general, *Proteobacteria* are considered to have negative impacts on gastrointestinal health, which, if a result of high levels of gastrointestinal iron, lignin would have beneficial effects. The physiological effects of these changes within the gastrointestinal tract would be an important future study, to confirm if these microbial changes lead to metabolomic changes.

Bacteroides (and specifically *Bacteroides uniformis*) were identified to be significantly elevated in the lignin with iron administration phase (Fig. 5Ei). This change could again be due to the enhanced bio-accessibility of iron conferred by lignin chelation. *Bacteroidaceae* have been found to be significantly enriched in participants undergoing iron supplementation.^38^ In studies where iron deprivation is invoked in the colon, *Bacteroidaceae* are found to diminish,^41^ including during the use of iron chelators.^42^ In this instance, lignin chelation and the increased iron bio-accessibility favoured bacterial growth. Many *Bacteroides* strains are recognised for their probiotic-like attributes, and in general, are considered beneficial in health.^43^ Furthermore, *Bacteroides* species have been recognised as potential next-generation probiotics, and thus, if lignin is acting as an indirect prebiotic, improving *Bacteroides* growth, this could be beneficial.^44^ It is evident that bacterial community changes are influenced by both iron and lignin supplementation. This could be due to the changes in iron solubility due to lignin supplementation as discussed but could be due to a pre-biotic effect conferred by lignin. It has long been considered that intestinal bacteria are unable to degrade lignin, with a general acknowledgement that lignin is resistant to degradation.^45^ However, recent reports suggest that lignin can be metabolised by specific bacterial strains,^46^ for example, *Lignolytica BRL6-1* (isolated from soil and able to metabolise lignin and its associated iron in growth studies).^47^

In summary, lignin demonstrates a strong iron-chelating ability which restricts intracellular iron acquisition. The lignin-iron complex itself is non-absorbable, and chelation within the murine gastrointestinal tract inhibited absorption with the bound iron lost in the faeces. Upon further examination in a colonic-bioreactor model, lignin-iron complexation led to increased iron solubility, yet this did not confer an increase in bio-accessibility. With respect to bacterial changes, lignin-iron chelation limited its utilisation by *Proteobacteria* yet favoured the growth of *Bacteroides*. This property of lignin could be useful in the context of inflammatory bowel disease where microbial dysbiosis is evident and patients are regularly supplemented with oral iron (and thus excess iron in the colon is present in the colon).^11^ This iron could exacerbate disease in the colon and contribute to the observed dysbiosis, where a colonic-targeted iron chelating agent with microbial altering activity such as lignin, would be beneficial.

## Author contributions

RDH, GOLD and CT conceptualised the study and developed the protocols and experiments. RDH and CT performed the statistical analysis. RDH, PP, ME and GOLD carried out the experiments. RDH, PP and ME analysed the data. RH and CT drafted the manuscript. All authors approved the manuscript.

## Conflicts of interest

The authors declare no conflicts of interest.

## Supplementary Material

FO-014-D2FO03807B-s001

FO-014-D2FO03807B-s002

FO-014-D2FO03807B-s003

FO-014-D2FO03807B-s004

FO-014-D2FO03807B-s005
