# Supplementary figures and images for: Intestinal iron bio-accessibility changes by Lignin and the subsequent impact on cell metabolism and intestinal microbiome communities
Source: Food Funct. 2023 Mar 21;14(8):3673–85. doi: 10.1039/d2fo03807b (PMC10123922; doi:10.1039/d2fo03807b)

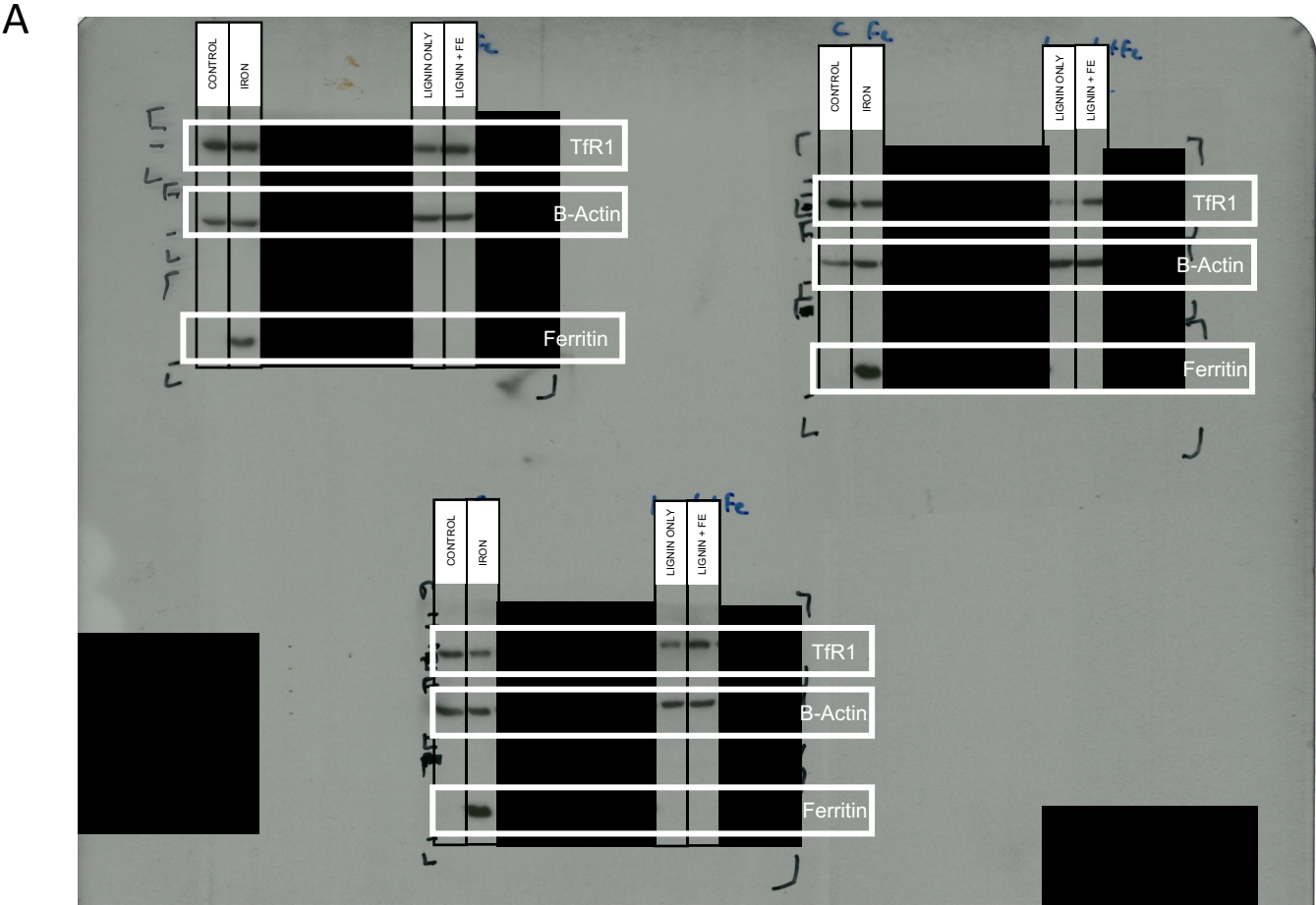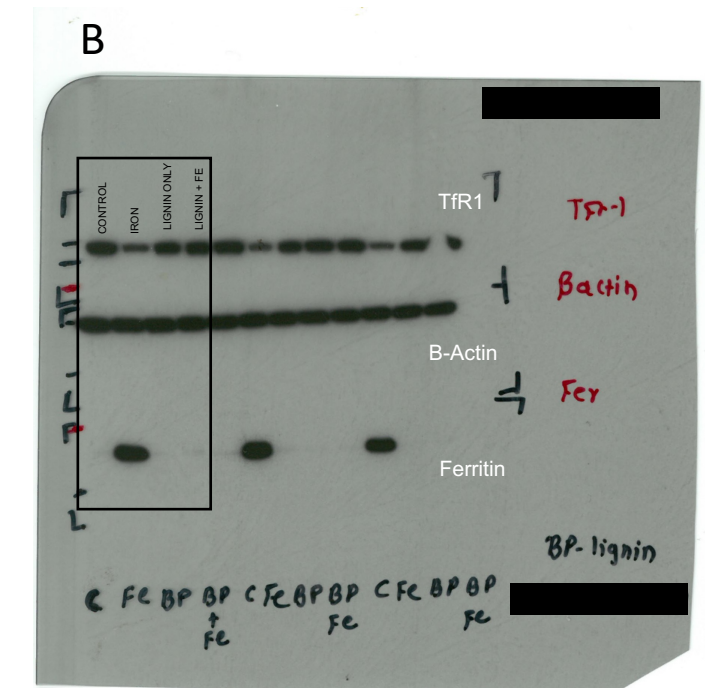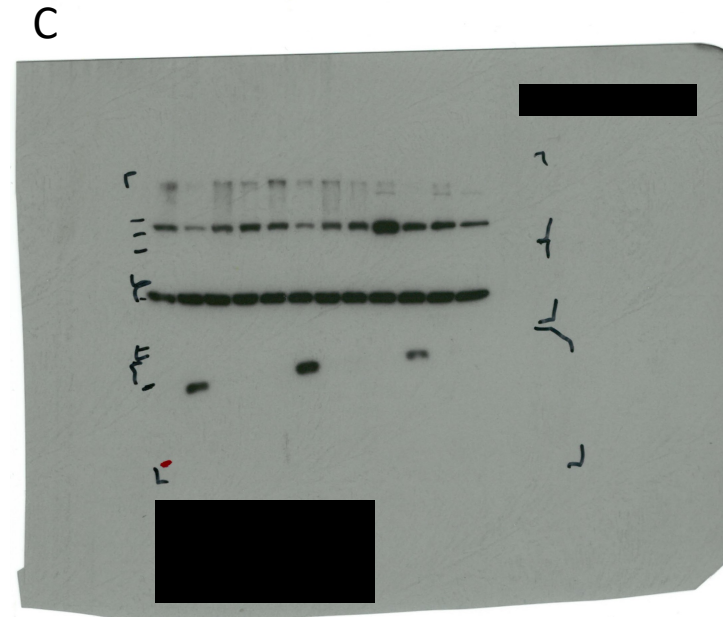

Supplement: FO-014-D2FO03807B-s001 [file FO-014-D2FO03807B-s001.pdf]

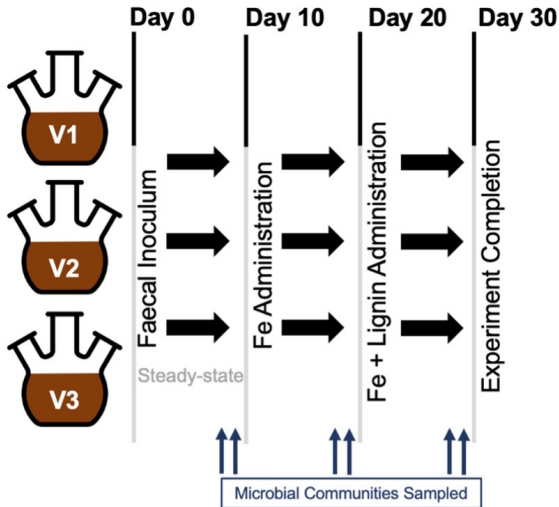

Supplement: FO-014-D2FO03807B-s004 [file FO-014-D2FO03807B-s004.pdf]
