# Supplementary material for: Intestinal iron bio-accessibility changes by Lignin and the subsequent impact on cell metabolism and intestinal microbiome communities
Source: Food Funct. 2023 Mar 21;14(8):3673–85. doi: 10.1039/d2fo03807b (PMC10123922; doi:10.1039/d2fo03807b)
Supplement: FO-014-D2FO03807B-s003 [file FO-014-D2FO03807B-s003.pdf]

Electronic Supplementary Material (ESI) for Food & Function.
This journal is © The Royal Society of Chemistry 2023

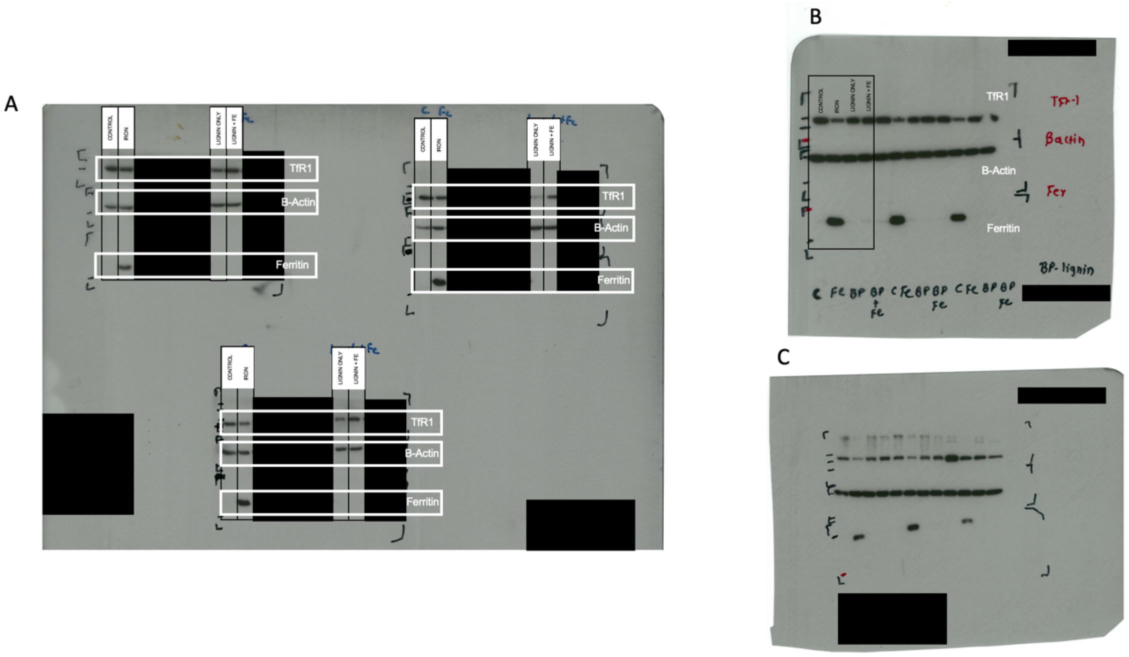

Full images of Western blots used to create Figure 2Ci and Cii. (A) Caco-2 cells non-treated (control), Fe-treated, Lignin treated and Lignin + Fe treated in lane 1,2 9 and 10 respectively (n=3 blots provided). **(B)** RKO cells non-treated (control), Fe-treated, Lignin treated and Lignin + Fe treated in lanes 1,2,3 and 4, repeated again in 5,6,7 and 8, repeated finally in 9,10,11 and 12 (thus n=3 provided). **(C)** Lower exposure Western blot identical to (B). TfR-1 = top band, B-actin = middle band and Ferritin = lower band.
