# Supplementary material for: Intestinal iron bio-accessibility changes by Lignin and the subsequent impact on cell metabolism and intestinal microbiome communities
Source: Food Funct. 2023 Mar 21;14(8):3673–85. doi: 10.1039/d2fo03807b (PMC10123922; doi:10.1039/d2fo03807b)
Supplement: FO-014-D2FO03807B-s005 [file FO-014-D2FO03807B-s005.pdf]

Electronic Supplementary Material (ESI) for Food & Function.
This journal is © The Royal Society of Chemistry 2023

1. SUPPLEMENTARY INFORMATION

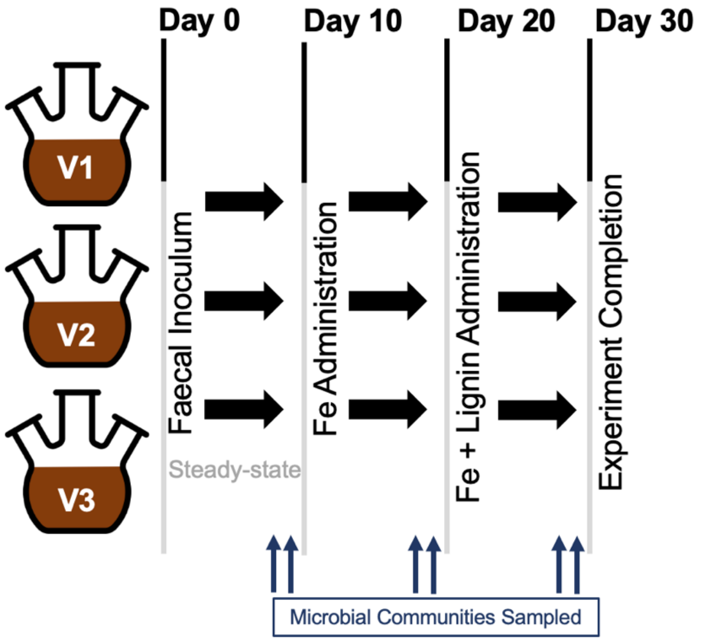

Schematic S1: Treatment schedule of the bioreactors. Three bioreactors (V1-3) were inoculated with a faecal microbiome on day 0. Following a 10-day equilibration period/steady-state phase *(referred to hereafter as the pre-supplementation phase),* $FeSO_4$ (10 mM) containing sodium ascorbate (50 mM) was introduced into the vessels to evolve the iron concentration within to 100 µM over three consecutive days. Thereafter, the iron concentration within the bioreactors was maintained at 100 µM *(referred to hereafter as the iron-supplementation phase).* Following this, lignin was also co-administered (with iron) by injecting an aqueous lignin solution to maintain bioreactor lignin concentrations at 0.3 % (w/v) in each vessel *(referred to hereafter as the lignin + iron supplementation phase).* Samples were taken throughout the experimental phases for iron analysis. For microbial community changes, samples were obtained at two time points at the end of each of the three phases (for example, day 8 and day 10 in each 10-day experimental phase period).

		**Unweighted UniFrac**		**Weighted UniFrac**	
**Group1**	**Group2**	Pseudo-F	p-value	Pseudo-F	p-value
Pre-Supplementation	Iron-only	3.274736	0.009	6.127362	0.013
Pre-Supplementation	Iron + Lignin	4.928861	0.003	29.179908	0.002
Iron-only	Iron + Lignin	1.522939	0.172	10.327314	0.001

Table S1: Pairwise PERMANOVA distance measurements results.
